# Optimization of Ultrasonic Parameters and Model Development for Nondestructive CTE Measurement of LAS Ultra-Low-Expansion Glass Ceramics

**DOI:** 10.3390/ma19183862

**Published:** 2026-09-10

**Authors:** Shuyun Chang, Wenqing Wei, Xue Qi, Xufeng Wang, Zuyi Zhang, Jian Gu, Dahong Mo, Hu Deng

**Affiliations:** 1 School of Information and Control Engineering, Southwest University of Science and Technology, Mianyang 621010, China; 18335550257@163.com (S.C.); wangxufeng2116@163.com (X.W.); 2 Innovation Center for Special Optical Glass Materials Technology, Chengdu 610100, China; zhangzy0318@126.com (Z.Z.); 18010584016@163.com (J.G.); modh@cdgmgd.com (D.M.); 3 College of Artificial Intelligence, Mianyang Teachers’ College, Mianyang 621000, China; 17378574923@163.com; 4 CDGM Glass Limited Liability Company, Chengdu 610100, China

**Keywords:** LAS glass ceramics, coefficient of thermal expansion, nondestructive inspection, longitudinal wave velocity, finite element method, ultrasonic CTE model

## Abstract

Lithium aluminosilicate (LAS) ultra-low-expansion glass ceramics are core materials for precision optical systems, whose quality and dimensional stability are critically constrained by the uniformity of the coefficient of thermal expansion (CTE). This work proposes a nondestructive ultrasonic immersion pulse reflection (UIPR) method for rapid and low-cost characterization of the CTE in LAS glass ceramics. Key parameters of the ultrasonic measurement system are optimized via finite element method (FEM) simulations and experimental validation. Employing the correlation method, the ultrasonic longitudinal wave velocity is measured in LAS glass ceramic samples with distinctly different CTE values. The proposed method achieves an ultrasonic longitudinal wave velocity measurement uncertainty of 0.49 m/s, contributing 4.78 ppb/°C to the overall uncertainty of ultrasonic CTE determination. Within the investigated sample set, a negative relationship is observed between ultrasonic longitudinal wave velocity and the mean CTE (0–50 °C). The linear fit yields a slope of −9.75736 (ppb/°C)/(m/s), with a Pearson correlation coefficient of −0.84303. Featuring noncontact and nondestructive capabilities, this method lays a solid methodological foundation for CTE evaluation and efficient iterative optimization of material fabrication processes. Meanwhile, it shows great potential for rapid full-aperture characterization of CTE uniformity in large-size LAS glass ceramics.

## 1. Introduction

Lithium aluminosilicate (LAS) glass ceramics are one of many advanced materials [1]. They are inorganic, nonporous lithium–aluminum–silicate crystalline materials featuring an ultra-low, near-zero coefficient of thermal expansion (CTE) [2]. Owing to their ultra-low thermal expansion properties and excellent dimensional stability [3], LAS glass ceramics have become a core material for large-aperture mirrors in astronomical applications [4]. During the fabrication of bulk LAS glass ceramics, the distribution uniformity of crystalline phases affects the internal CTE uniformity of the material and significantly influences the forming quality of mirror blanks, as well as the imaging stability of mirror surfaces after optical processing [5]. Accordingly, measuring the CTE and its distribution uniformity is of critical importance. To date, various methodologies have been developed to measure the CTE of materials, with linear variable differential transformer (LVDT) [6], optical interferometer [7], and linear incremental encoder [8] methods being the three dominant approaches for CTE characterization of LAS glass ceramics. Jedamzik et al. periodically calibrated an improved laser dilatometer with titanium silicate reference samples, exhibiting an absolute accuracy of 0.5 ppb/°C, enabling an overall precision of ±6.2 ppb/°C for CTE (0–50 °C) measurements with a repeatability of ±1.2 ppb/°C—four times better than that of standard LVDT dilatometers [7]. In 2015, the research team developed an advanced dilatometer based on linear incremental encoders for high-precision displacement measurement. The instrument achieved a measurement accuracy of ±3 ppb/°C with a repeatability of ±1 ppb/°C [8,9].

Although the aforementioned CTE measurement methods are characterized by high precision and broad applicability, they still present notable limitations that warrant further investigation. Specifically, dilatometers require periodic system calibration with standard reference samples. The testing procedure is inherently destructive and sampling-based, with long measurement cycles and high costs [10]. Most critically, they cannot perform rapid, full-aperture nondestructive CTE characterization of large LAS glass ceramics. Ultrasonic technology, as a nondestructive and cost-effective approach to determining CTE in large and ultra-large glass components, exhibits remarkable application potential [11]. Hagy et al. investigated full-aperture detection of CTE and its uniformity for large-size bulk ultra-low-expansion fused silica glass (ULE) using an ultrasonic method and obtained a set of important research results [12]. Furthermore, Wei et al. established the inter-relationships among the Young’s modulus, longitudinal wave velocity, and CTE, using the Young’s modulus as an intermediate variable. Based on this relationship, they conducted full-aperture ultrasonic testing to investigate the CTE and its uniformity in large-sized ULE [13,14]. They pointed out that the key challenges of this technique lie in the high-precision measurement of the longitudinal wave velocity in materials and the accurate establishment of an ultrasonic CTE measurement model [15]. Considering that both LAS glass ceramics and ULE have a glassy phase and similar ultra-low CTE, it is feasible to determine their CTE through ultrasonic nondestructive testing.

Although LAS glass ceramics represent key materials for precision optical systems, their quality and dimensional stability are frequently limited by the uniformity of CTE [16]. In this study, we propose an ultrasonic immersion pulse reflection (UIPR) method, in which samples are immersed in water and the sample acoustic velocity is obtained from ultrasonic echo signals, featuring fast and accurate velocity measurement. This approach enables the establishment of a high-precision ultrasonic CTE measurement model using CTE calibration data from 50 mm long samples. The mean CTE (0–50 °C) of LAS glass ceramic samples with markedly different CTE values is determined using a laser dilatometer. Their corresponding ultrasonic longitudinal wave velocity (*c*_L_) values are determined via a correlation algorithm. The relationship between the mean CTE over the temperature range of 0–50 °C (denoted as *α*_m_) and *c*_L_ is investigated experimentally. The obtained results are discussed, and a comprehensive uncertainty analysis is performed to identify pathways for developing higher-precision measurement techniques and equipment.

## 2. Materials and Methods

### 2.1. Principles and Method for Measurement of the Ultrasonic Velocity

LAS glass ceramics are a class of glass ceramic materials fabricated by subjecting shaped Li_2_O–Al_2_O_3_–SiO_2_ system parent glasses to nucleation and crystallization via heat treatment, where the parent glasses are initially prepared using the melt-quenching method. Note that the resulting CTE strongly depends on the applied heat treatment procedures, which are not fixed [17]. The samples investigated in this work derive from one batch of LAS glass ceramics produced under a specific heat treatment regime [18]. The amorphous LAS base glass is subjected to two-stage heat treatment for nucleation and crystallization; specifically, the samples were heated to 695 °C at a heating rate of 20 °C/h under an ambient air atmosphere and held at this temperature for a certain duration for nucleation. Subsequently, the temperature was increased to 770–790 °C at a heating rate of 10 °C/h, followed by another isothermal hold for crystallization. After crystallization, the samples were cooled to 120 °C at a cooling rate of 30 °C/h, then furnace-cooled to room temperature [19]. The type, content, and grain size of the crystalline phases collectively determine the CTE of the material, and also jointly influence the variation in the Young’s modulus *E*, while exerting negligible effects on density *ρ* and Poisson’s ratio *μ*. For isotropic solid materials, the relationship between *c*_L_ and the Young’s modulus *E* is given as follows [14]:

(1) $$c_{L}={\left[\frac{E(1-\mu )}{\rho (1+\mu )(1-2\mu )}\right]}^{\frac{1}{2}},$$

The foregoing analysis indicates a monotonic coupling between *c*_L_ and CTE. For LAS glass ceramics with fixed thickness, CTE can be determined nondestructively by measuring the ultrasonic time of flight (TOF), based on the observed approximately linear trend within the investigated sample range. To improve the accuracy of ultrasonic-based CTE measurement, this work proposes a four-step technical strategy: (1) selecting LAS glass ceramic samples with distinctly different CTE values; (2) precisely determining the *c*_L_ using the proposed method; (3) performing high-precision CTE calibration; and (4) establishing an ultrasonic CTE measurement model.

To accurately determine the ultrasonic velocity in LAS glass ceramics, the ultrasonic immersion pulse reflection (UIPR) method is adopted based on available experimental conditions and material characteristics. This method generally involves performing time-domain analysis based on the TOF principle. The most straightforward approach to obtaining the TOF is derived from the ratio of the material thickness *d* to the ultrasonic propagation time ∆*t* [20,21].

**Figure 1:**
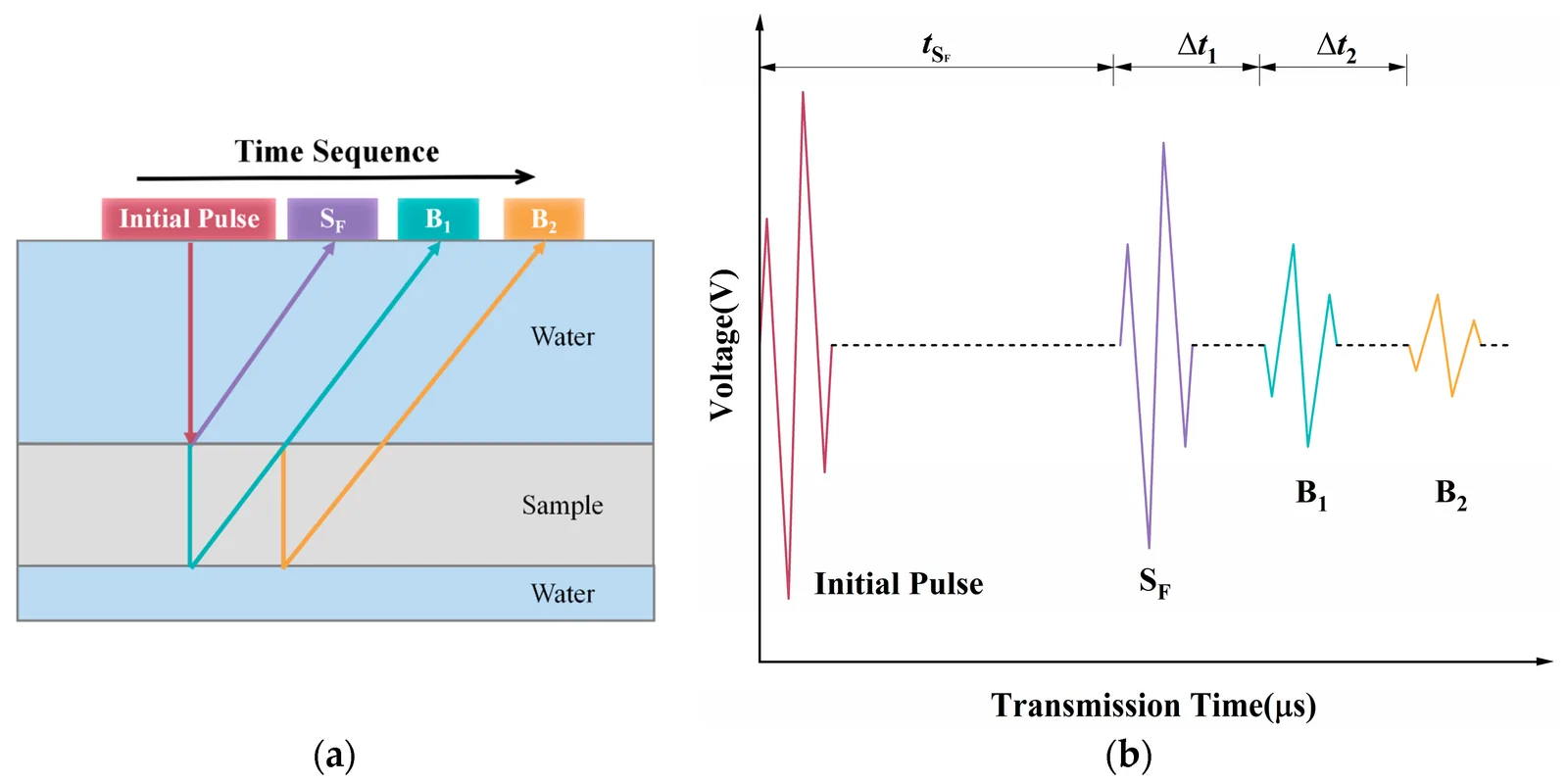
Principle of the ultrasonic immersion pulse reflection method: (**a**) ultrasonic wave propagation process in the UIPR method; (**b**) ultrasonic wave propagation time in the UIPR method.

Considering the material properties, geometry, and dimensions of the test samples, a highly integrated UIPR-based *c*_L_ measurement system was developed in this work. The UIPR method operates on the following principle: when ultrasonic waves impinge on an interface between media with different acoustic impedances, reflection and transmission occur simultaneously. The front surface echo is denoted as S_F_, the primary bottom echo as B_1_, and the secondary bottom echo as B_2_, as illustrated in Figure 1a. Owing to the high waveform similarity between B_1_ and B_2_, they can be readily identified from the signals acquired by the transducer, and the TOF can be extracted from the time interval ∆*t*_2_ between B_1_ and B_2_.

(2) $$c_{L}=2d/\Delta t_{2}.$$

According to Equation (2), *c*_L_ can be calculated by determining ∆*t*_2_ together with the measured *d* value.

### 2.2. Method for CTE Calibration

A laser interferometric dilatometer (LINSEIS, L75 laser, Vielitzerstr, Selb, Germany) equipped with a frequency-stabilized He–Ne laser (center wavelength: 632.8 nm, output power: 2 mW) was selected for the calibration of *α*_m_, leveraging its advantages of noncontact measurement and high accuracy. It should be emphasized that CTE measurement performed with this dilatometer is destructive and cannot be performed in an online, in situ manner. Accordingly, it is only utilized in this work as a reference validation approach for constructing the ultrasonic CTE measurement model.

The axial length change in the sample ∆*L* induced by a temperature variation Δ*T* is expressed as

(3) $$\Delta L=L_{0}\alpha_{m}(T_{2}-T_{1})=L_{0}\alpha_{m}\Delta T,$$

where *L*_0_ denotes the initial length of the sample.

The incident laser beam is split into two beams after propagating through a semi-reflective lens. One beam is irradiated onto the end face of the sample, while the other is directed onto the reference end face. Upon reflection, the two beams recombine and generate interference, and the resulting optical path difference (OPD) depends exclusively on Δ*L* [22]. The value of αm can be calculated by recording the variations in OPD and temperature, as follows:

(4) $$\alpha_{m}=\Delta L/(L_{0}\Delta T)=\lambda \Delta N/(2L_{0}\Delta T),$$

where *λ* is the laser wavelength and Δ*N* is the change in the number of interference fringes.

## 3. Sample Preparation and Experimental Setup

### 3.1. Sample Preparation and Experimental Setup for the c_L_ Measurement

**Figure 2:**
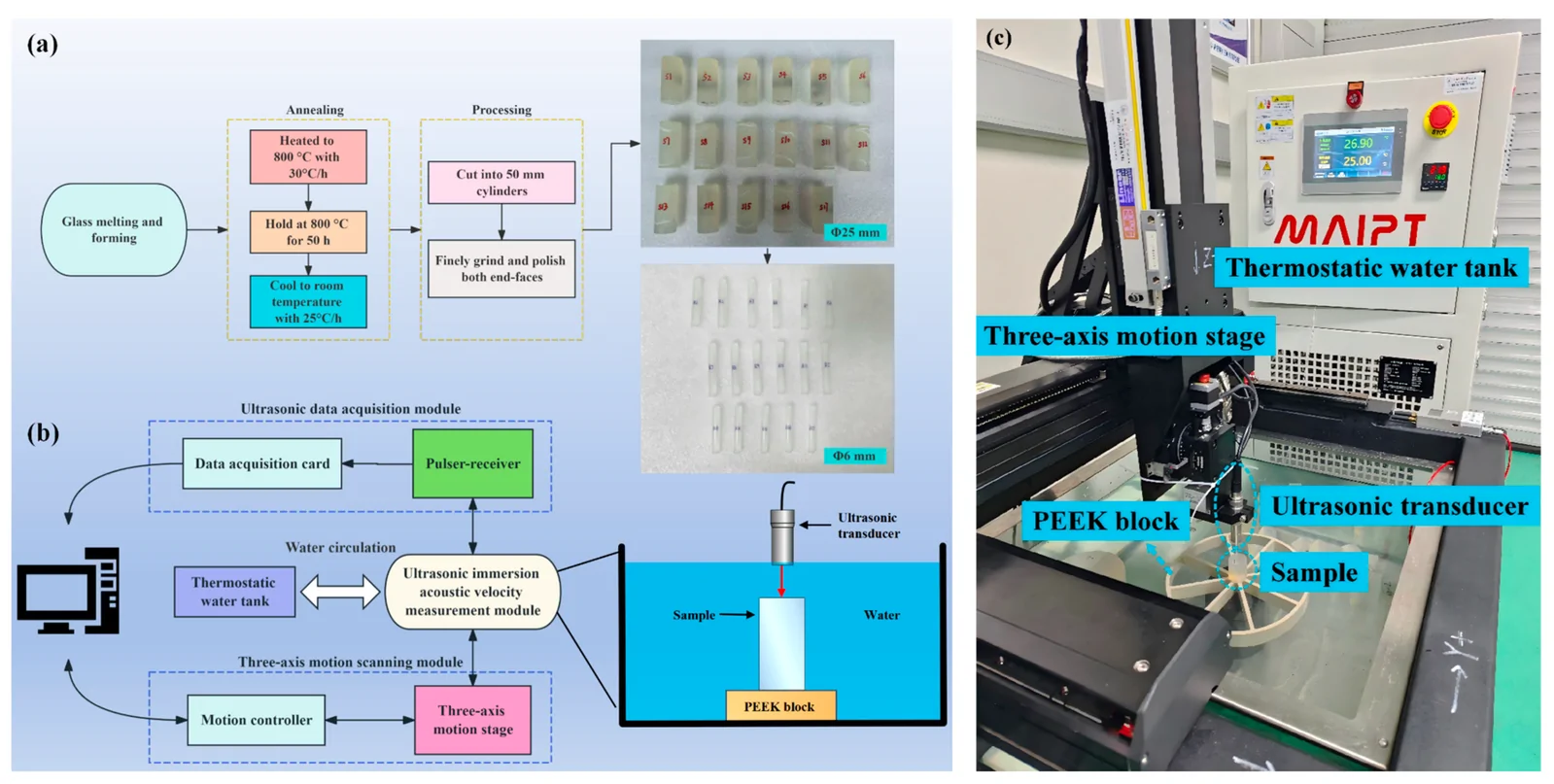
Sample preparation and experimental setup for *c*_L_ measurement: (**a**) sample preparation procedure; (**b**) general layout of the UIPR-type *c*_L_ measurement system; (**c**) photograph of the experimental setup.

The selected material was LAS glass ceramic, which was first annealed to remove internal residual thermal stresses before furnace discharge. After annealing, the residual stress of all samples was lower than 10 nm/cm, with a maximum value of 2.329 nm/cm, indicating a low-stress state. The following two specifications are required for preparing LAS glass ceramic samples: (1) To ensure consistency between the samples for ultrasonic velocity measurement and CTE calibration, the sample dimensions must satisfy the requirements for CTE calibration. Specifically, 17 glass ceramic rod samples were cut from bulk raw blanks using a high-speed water-jet cutting process and prepared in a cylindrical geometry with approximate dimensions of Φ25 mm × 50 mm. (2) The two end faces of the test samples must be flat and parallel to each other; thus, these samples were finely ground and polished via rough polishing followed by fine polishing steps to achieve a flatness of 0.5*λ* (*λ* = 632.8 nm) and a parallelism of 20 μm for *c*_L_ measurement. The prepared samples were numbered S1–S17 (Figure 2a).

CTE calibration is based on Michelson interference, thus imposing strict requirements on the samples: (1) the sample should be cylindrical, with dimensions Φ6 mm × 50 mm; (2) the two end faces must be flat and parallel to each other; and (3) the roundness and taper of the sample should be controlled according to general standards. First, the cylindrical samples were cut using a high-speed water jet at the center of the above *c*_L_ measurement samples, and the thickness was kept as close as possible to 50 mm to avoid the random error caused by the poor clarity of the interference fringe. The two end faces of the cut samples were then ground and polished. Finally, the perpendicularity was controlled. The final prepared samples are shown in Figure 2a, and their specific parameters are given in Table 1 [23].

A schematic diagram of the *c*_L_ measurement system is shown in Figure 2b. The system consists of an ultrasonic pulser–receiver, an immersion ultrasonic transducer, a data acquisition card (M4i.2210-x8, Spectrum Instrumentation GmbH, Grosshansdorf, Germany), an immersion coupling device, a thermostatic water tank, a three-axis motion scanning module, and a personal computer (PC). Inside the water tank of this immersion coupling device sits a PEEK (full name is Polyether Ether Ketone) block that acts as the sample holder. The acoustic impedance of PEEK lies at a moderate level between those of water and LAS glass ceramics. While providing stable mechanical support for the samples, this configuration effectively suppresses echo interference from the base block and ensures high-accuracy ultrasonic velocity measurement. The full water-immersion coupling scheme adopted in this system provides more stable ultrasonic signal coupling, compared with the direct-contact method. The basis for device selection and parameter configuration of key components in this system is as follows.

High-precision TOF measurements require a receiving system with a fast response and broadband frequency response to ensure sufficient temporal and depth resolution. In this study, a single-point sampling strategy was adopted to determine the longitudinal wave velocity *c*_L_ of LAS glass ceramics. To satisfy the requirements of pulse excitation and reception, a spike-pulse pulser–receiver (JSR, DPR300, Pittsford, NY, USA) with fast response and wideband performance was selected.

### 3.2. Optimization of the Ultrasonic Transducer

The performance of ultrasonic inspection is jointly affected by the operating acoustic frequency of the transducer and the diameter of its piezoelectric chip. The basic principles used for ultrasonic parameter selection are as follows. First, the two echo signals B_1_ and B_2_ must be clearly distinguishable with a correlation coefficient higher than 0.995. Second, the measured ultrasonic velocity should exhibit good consistency and a low standard deviation. Guided by these two principles, numerical simulations and experimental measurements were designed and carried out in this study to optimize the ultrasonic parameters.

#### 3.2.1. Simulation Study on Effects of Different Ultrasonic Transducer Configurations on Ultrasonic Velocity in LAS Glass Ceramics

**Figure 3:**
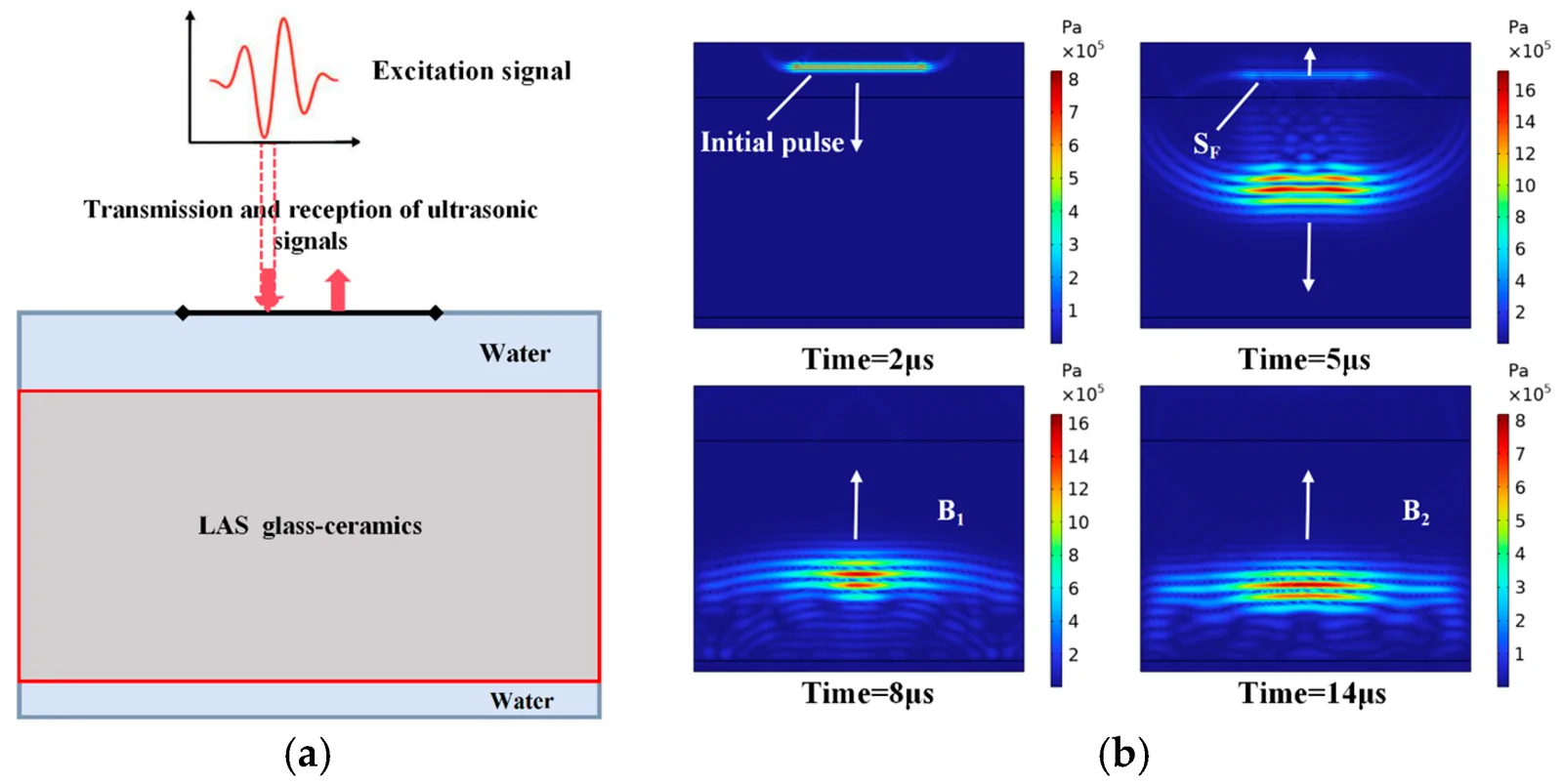
Finite element simulation of ultrasonic wave propagation in LAS glass ceramics: (**a**) simulation model of ultrasonic immersion pulse-echo measurement; (**b**) acoustic wave propagation at different times.

In the COMSOL software (version 6.3), a finite element simulation model was established to compare the ultrasonic velocity measurement performance of ultrasonic transducers with different parameters for LAS glass ceramics. The parameter settings of the finite-element simulation model are described as follows. For the LAS glass ceramic domain, the width and height were set to 30 mm and 20 mm, respectively, with 5 mm and 1 mm water layers assigned at the top and bottom boundaries. Free triangular meshes were adopted, and the mesh size was specified as 1/5 of the wavelength in water. A uniform finite-element solving time step of 2 ns was applied. The ultrasonic excitation source was simulated using a sinusoidal oscillation signal modulated by a Hanning window, as shown in Equation (5). The duration of the Hanning window was set to three fundamental frequency periods, and its central frequency was denoted as *f*_0_. The simulation model is illustrated in Figure 3a, and the corresponding model parameters are summarized in Table 2.

(5) $$F(t)=-0.5[1-\cos(\frac{2\pi f_{0}t}{3})]\sin(2\pi f_{0}t).$$

The simulations were conducted in strict accordance with the existing and available water-immersion planar ultrasonic transducers in the laboratory, covering typical frequency–diameter combinations to systematically analyze the response characteristics of the transducers. The propagation of ultrasonic waves at different time instants in the simulation is shown in Figure 3b.

Ultrasonic simulations were performed for transducers with different frequencies and piezoelectric wafer diameters. The resulting ultrasonic velocity accuracy and apparent signal attenuation characteristics are summarized in Table 3.

Based on the data in Table 3, the simulation results are systematically analyzed and interpreted with respect to two key parameters: apparent attenuation and relative ultrasonic velocity deviation. This analysis evaluates the performance of different transducer configurations for ultrasonic velocity measurement in LAS glass ceramics and provides theoretical support and guidance for the selection of experimental setups in subsequent work.

Apparent attenuation (dB) quantifies the degree of signal attenuation as ultrasonic waves propagate through the material. A more negative attenuation value corresponds to more severe signal loss, which may degrade the inspection signal-to-noise ratio (SNR) and signal clarity. The relative ultrasonic velocity deviation describes the discrepancy between simulated and theoretical ultrasonic velocity; a smaller deviation indicates that the simulation model more closely matches real physical conditions.

The simulation results reveal notable differences in ultrasonic velocity measurement accuracy and signal attenuation characteristics among transducers with different frequencies and element diameters. In terms of relative ultrasonic velocity deviation, medium-frequency transducers exhibit higher simulation accuracy and offer good reliability for practical ultrasonic velocity inspection. The ultrasonic velocity deviation tends to increase with rising frequency or decreasing element diameter. For example, the 15 MHz–6 mm transducer yields a deviation of 0.19%, reflecting that high-frequency, small-diameter transducers are more sensitive to the acoustic properties of the material in simulations and are more strongly influenced by interfacial scattering and dispersion effects in the model.

Meanwhile, the simulation data show that attenuation intensifies with increasing frequency, and transducers with smaller diameters exhibit more pronounced attenuation at the same frequencies. For instance, the 5 MHz–13 mm transducer has an apparent attenuation of −3.7 dB, which is similar to the results of other diameter configurations at the same frequency, indicating that diameter has a limited effect on attenuation in the low- to medium-frequency range. In contrast, the 10 MHz–6 mm and 15 MHz–6 mm transducers reach attenuation values of −7.0 dB and −7.7 dB, respectively. This suggests that high-frequency small-diameter transducers experience significant acoustic energy loss when penetrating glass ceramics with multiple crystalline phases, which is mainly attributed to enhanced scattering and absorption of ultrasonic waves in the crystal–glass composite structure. Therefore, to maintain a favorable SNR while ensuring a certain inspection depth, the configuration combining medium frequency with a relatively large element diameter demonstrates superior signal retention performance.

Overall, the above simulation data confirm that medium-frequency transducers with large diameters achieve well-balanced performance between ultrasonic velocity deviation and signal attenuation. With high ultrasonic velocity accuracy and relatively low attenuation, they enable stable and repeatable ultrasonic velocity measurement in materials with complex phase compositions such as LAS glass ceramics. These results provide a direct basis for transducer selection in subsequent experiments. Additionally, appropriately increasing the element diameter within the medium-frequency range helps to suppress attenuation and improves the recognizability of inspection signals, which offers clear guidance for achieving high-precision ultrasonic velocity measurement in LAS glass ceramics.

#### 3.2.2. An Experimental Study of the Effect of Different Ultrasonic Transducer Configurations on the Ultrasonic Velocity of LAS Glass Ceramics

**Figure 4:**
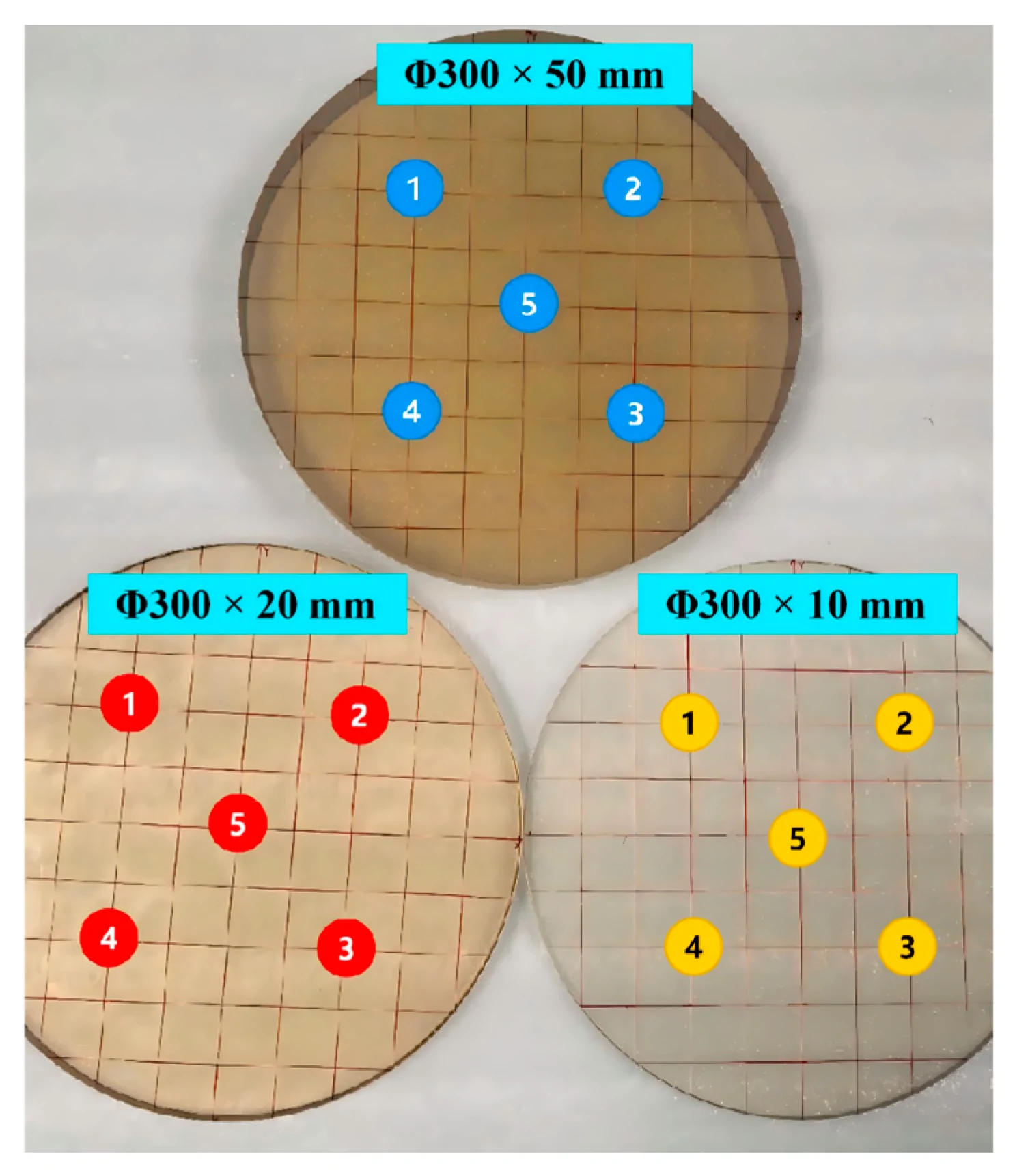
Three tested samples of LAS glass ceramics with different thicknesses.

Based on the aforementioned simulation results, experimental measurements were performed on three LAS glass ceramic samples of varying thicknesses (Φ300 mm × 10 mm, Φ300 mm × 20 mm, and Φ300 mm × 50 mm, as shown in Figure 4) using ultrasonic transducers with different frequencies and element diameters.

For each sample, five representative measurement points (1, 2, 3, 4, and 5) were selected for ultrasonic velocity testing. Five replicate measurements were conducted at each point, and the average value was adopted as the final measurement result.

**Figure 5:**
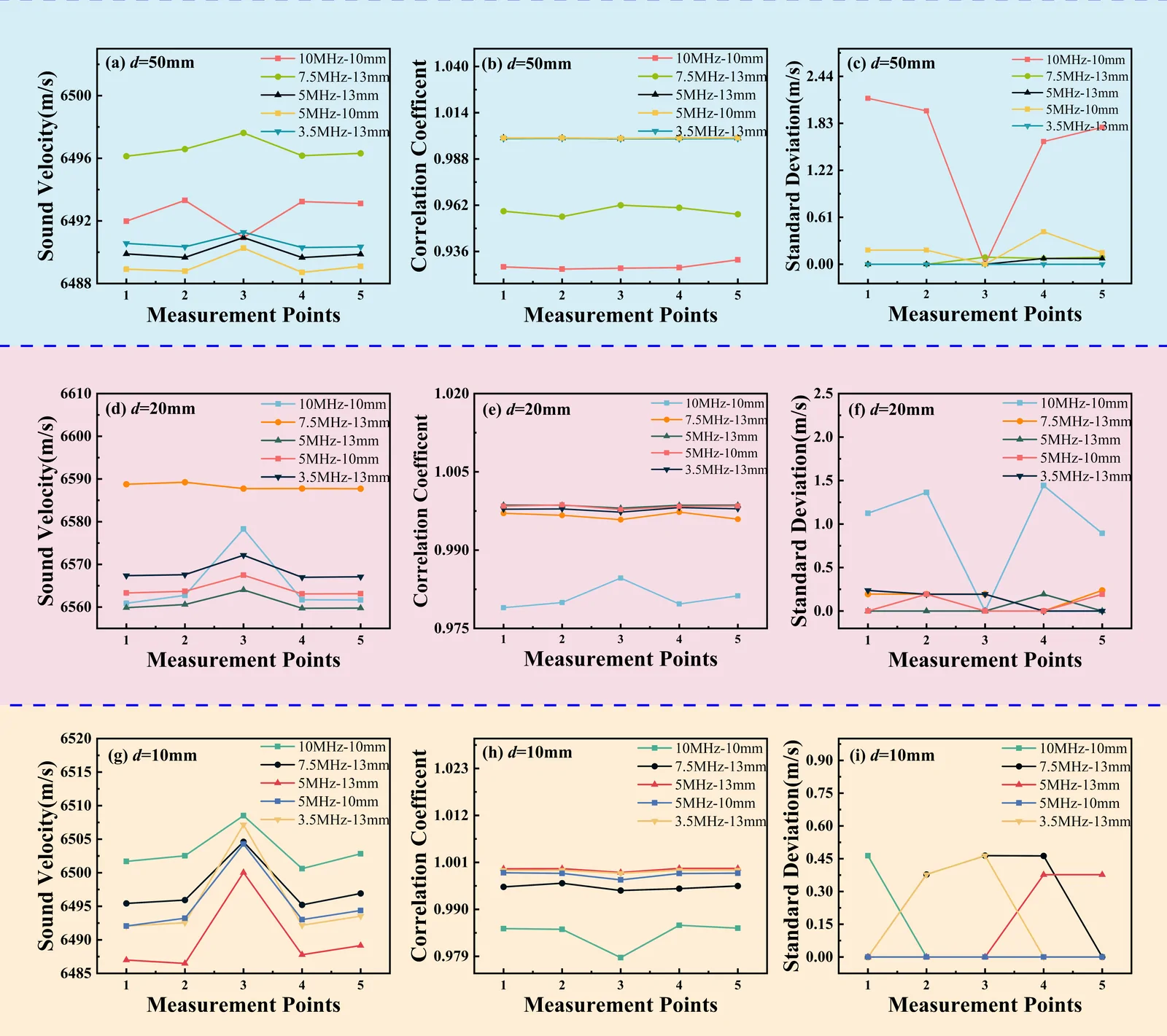
Comprehensive comparative chart of measurement results obtained with different ultrasonic transducers: (**a**–**c**) measurement results for samples with thicknesses of 50 mm; (**d**–**f**) measurement results for samples with thicknesses of 20 mm; (**g**–**i**) measurement results for samples with thicknesses of 10 mm.

As shown in Figure 5, the measurement results obtained with different transducer configurations exhibit distinct differences. These results are based on repeated ultrasonic velocity measurements conducted at five randomly selected points on three LAS glass ceramic samples with thicknesses of 10 mm, 20 mm, and 50 mm, respectively.

In terms of the proximity of the measured ultrasonic velocity values to the material’s theoretical ultrasonic velocity, the 5 MHz–13 mm and 5 MHz–10 mm transducers yield results closest to the theoretical value. Among them, the 5 MHz–13 mm transducer achieves the highest measurement consistency, with the smallest average ultrasonic velocity deviation across samples of all thicknesses, indicating that this configuration possesses excellent accuracy and material adaptability in practical measurements.

Further analysis of signal stability and data processing reliability shows that when ultrasonic velocity is calculated via the correlation method, the 5 MHz–13 mm, 5 MHz–10 mm, and 3.5 MHz–13 mm transducers all maintain high correlation coefficients in the range of 0.998–0.999. This reflects good consistency of their time-domain waveforms and high credibility of the calculated ultrasonic velocity values. In addition, the standard deviations of ultrasonic velocity from five replicate measurements at each point for these three transducers remain within a low range, demonstrating outstanding measurement repeatability and further confirming their favorable stability and anti-interference capability in practical inspection.

Overall, considering the three evaluation metrics of ultrasonic velocity accuracy, correlation coefficient, and measurement repeatability, the 5 MHz–13 mm transducer delivers nearly optimal performance across all indicators—it not only ensures ultrasonic velocity measurements close to the theoretical value, but also features high signal consistency and low fluctuation, making it suitable for precise acoustic inspection of materials with multiphase composite structures such as LAS glass ceramics.

Accordingly, the 5 MHz–13 mm transducer was ultimately selected as the inspection transducer for subsequent systematic experiments in this work. This selection is consistent with the conclusions of the prior simulation analysis, which verifies the effectiveness of numerical simulations in guiding experimental configuration design.

## 4. Results and Discussion

### 4.1. c_L_ Measurement

The experimental procedure included the following five parts.

(1) Thickness measurement

The thickness was measured via a 25–50 mm micrometer with a precision of 1 μm. The measured *d* value was taken as the average of five measurements to reduce the influence of random errors.

(2) Sample placement and temperature control

The coupling setup for *c*_L_ measurement is illustrated in Figure 2. First, the test sample was centered on the PEEK support block positioned at the center of the water tank, ensuring coaxial alignment between the transducer and the sample with their centers perfectly matched. The entire test platform was then immersed in the water tank.

The water temperature was maintained at 25 °C and monitored via a thermometer to mitigate the influence of temperature fluctuations on *c*_L_ measurement results. After the sample was allowed to equilibrate for 120 min to reach thermal equilibrium, multiple sets of ultrasonic echo signals were acquired using a PC.

(3) Optimization of the water distance and gain

The water distance, denoted as *H*, refers to the distance from the transducer end-face to the upper surface of the sample in water. Let *d* be the thickness of the sample. To ensure that the second surface echo appears after the primary bottom echo and avoid interference with signal identification, *H* needs to satisfy

(6) $$H>\frac{c_{w}}{c_{s}}d,$$

where *c*_w_ is the acoustic velocity in water and *c*_s_ is the classical acoustic velocity of the sample.

**Figure 6:**
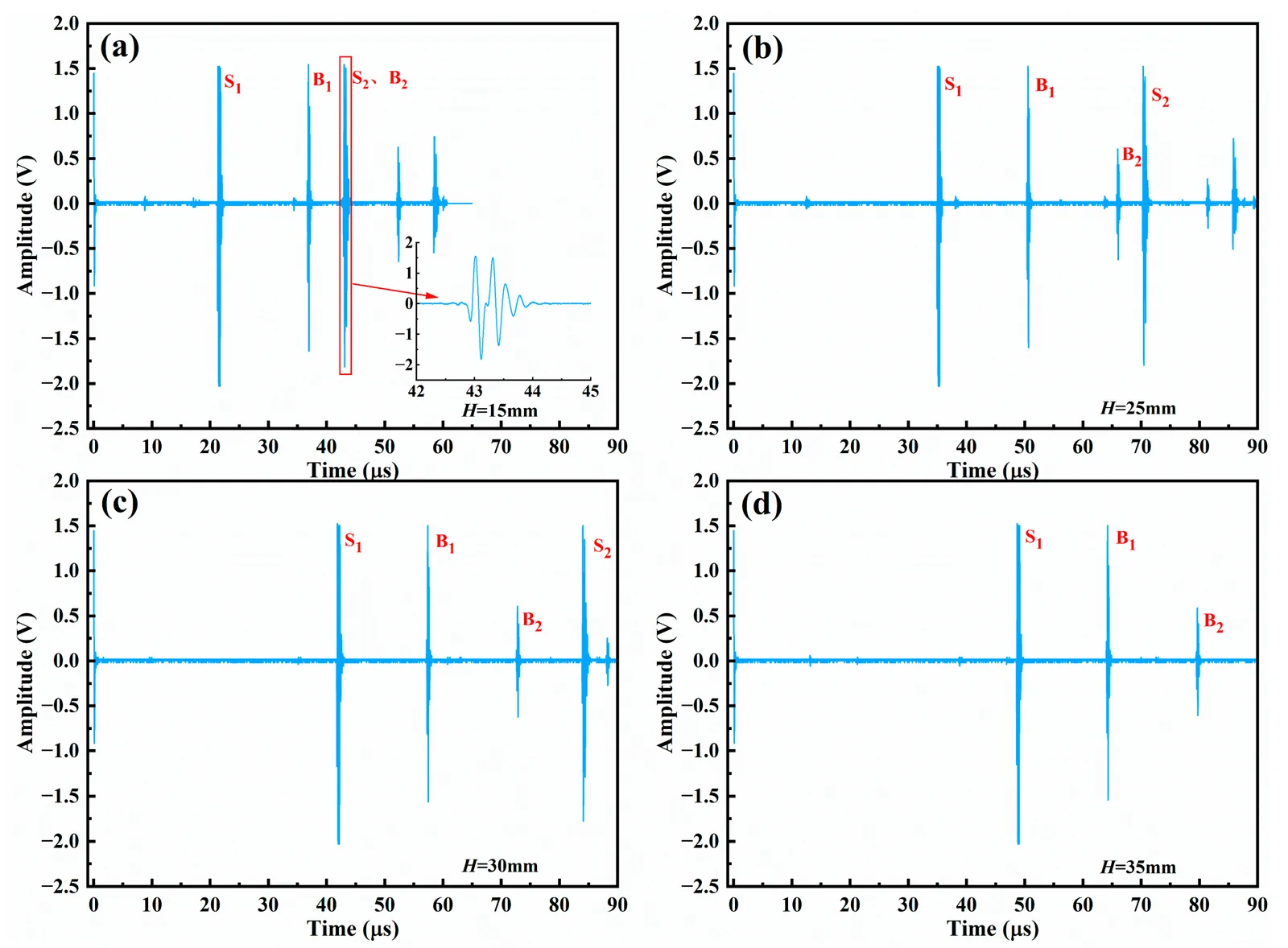
Ultrasonic echo signals at different *H* values: (**a**) *H* = 15 mm; (**b**) *H* = 25 mm; (**c**) *H* = 30 mm; (**d**) *H* = 35 mm.

Based on the thickness (50 mm) of the sample used for model establishment, together with the classical acoustic velocity values of water (1497 m/s at 25 °C) and LAS glass ceramics (6486 m/s), the water path *H* was calculated to be greater than 11.54 mm. The Z-axis of the three-axis motion system was then used to adjust *H* to the range of 15–35 mm, and echo signals of the above sample corresponding to four different *H* values were acquired under a constant gain of 31 dB, as shown in Figure 6. Herein, S_1_ and S_2_ represent the first and second surface echoes (S_F_), respectively.

As can be observed from Figure 6, for the 50 mm thick LAS glass ceramic sample, the secondary surface echo S_2_ overlaps with the secondary bottom echo B_2_ in the acquired ultrasonic signals at a water path of 15 mm, making B_1_ and B_2_ indistinguishable. When the water path ranges from 25 mm to 30 mm, the S_2_ signal gradually separates from the B_2_ echo. At a water path of 35 mm, only S_1_, B_1_, and B_2_ are present in the acquired signals, which greatly facilitates the identification of B_1_ and B_2_. In summary, with comprehensive consideration of detection sensitivity and measurement accuracy, the optimal water path for the 50 mm LAS glass ceramic sample was determined to be 35 mm.

After determining the optimal water path, the gain parameter was set based on the waveforms and amplitude attenuation characteristics of the B_1_ and B_2_ signals.

**Figure 7:**
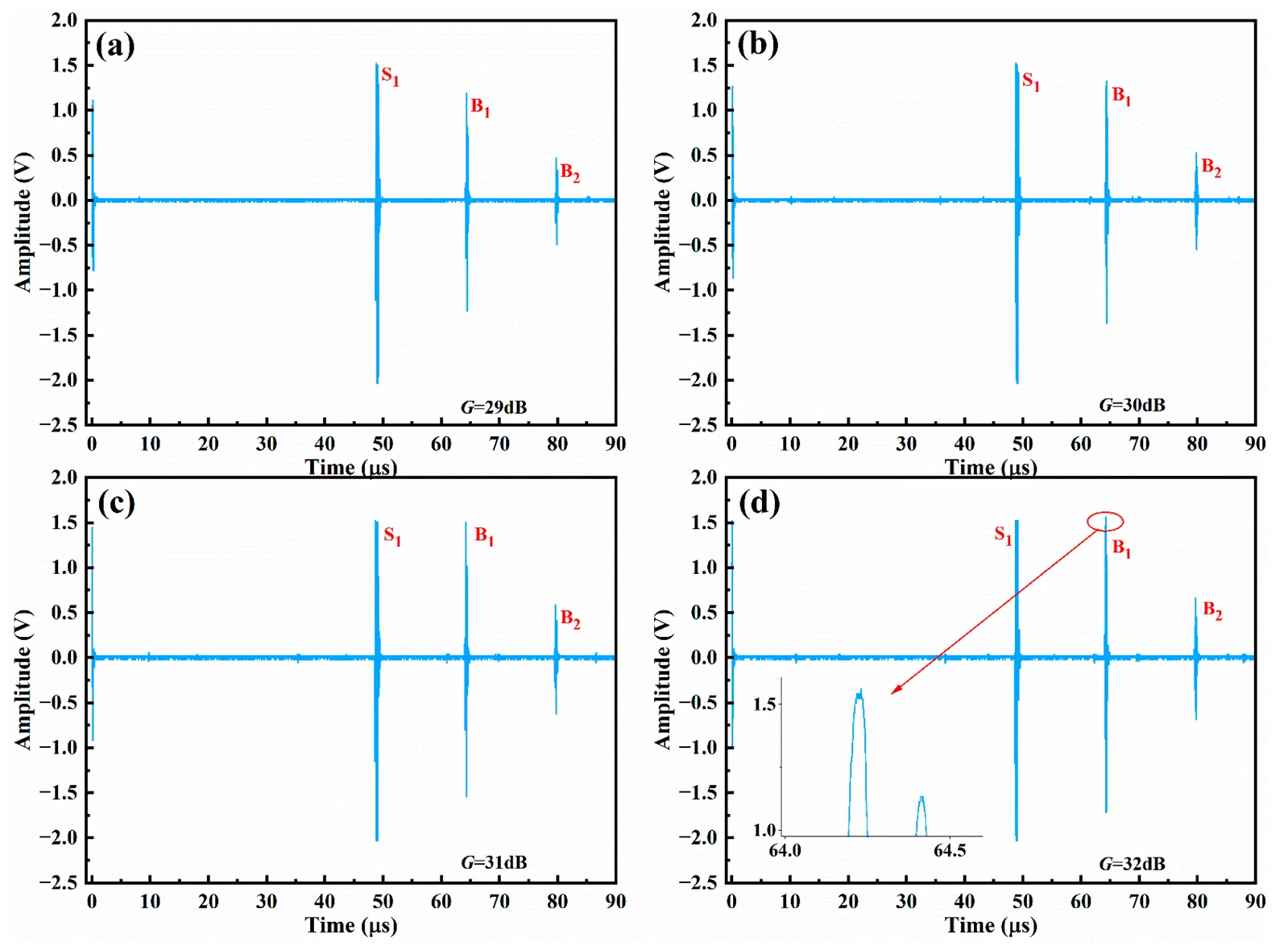
Ultrasonic echo signals at different gains: (**a**) 29 dB; (**b**) 30 dB; (**c**) 31 dB; (**d**) 32 dB.

Four gain levels (29 dB, 30 dB, 31 dB, and 32 dB) were applied to the 50 mm LAS glass ceramic sample. As presented in Figure 7, the amplitudes of the B_1_ and B_2_ signals show significant attenuation at a gain of 29 dB. With the gradual increase in gain, the amplitudes of the B_1_ and B_2_ signals rise correspondingly. When the gain reaches 32 dB, signal distortion occurs in the B_1_ echo, meaning the gain cannot be further increased. Therefore, integrating the waveforms and amplitude attenuation performance of B_1_ and B_2_ signals, the optimal gain for the 50 mm LAS glass ceramic sample was selected as 31 dB.

(4) Acquisition of the ultrasonic signals and determination of TOF

**Figure 8:**
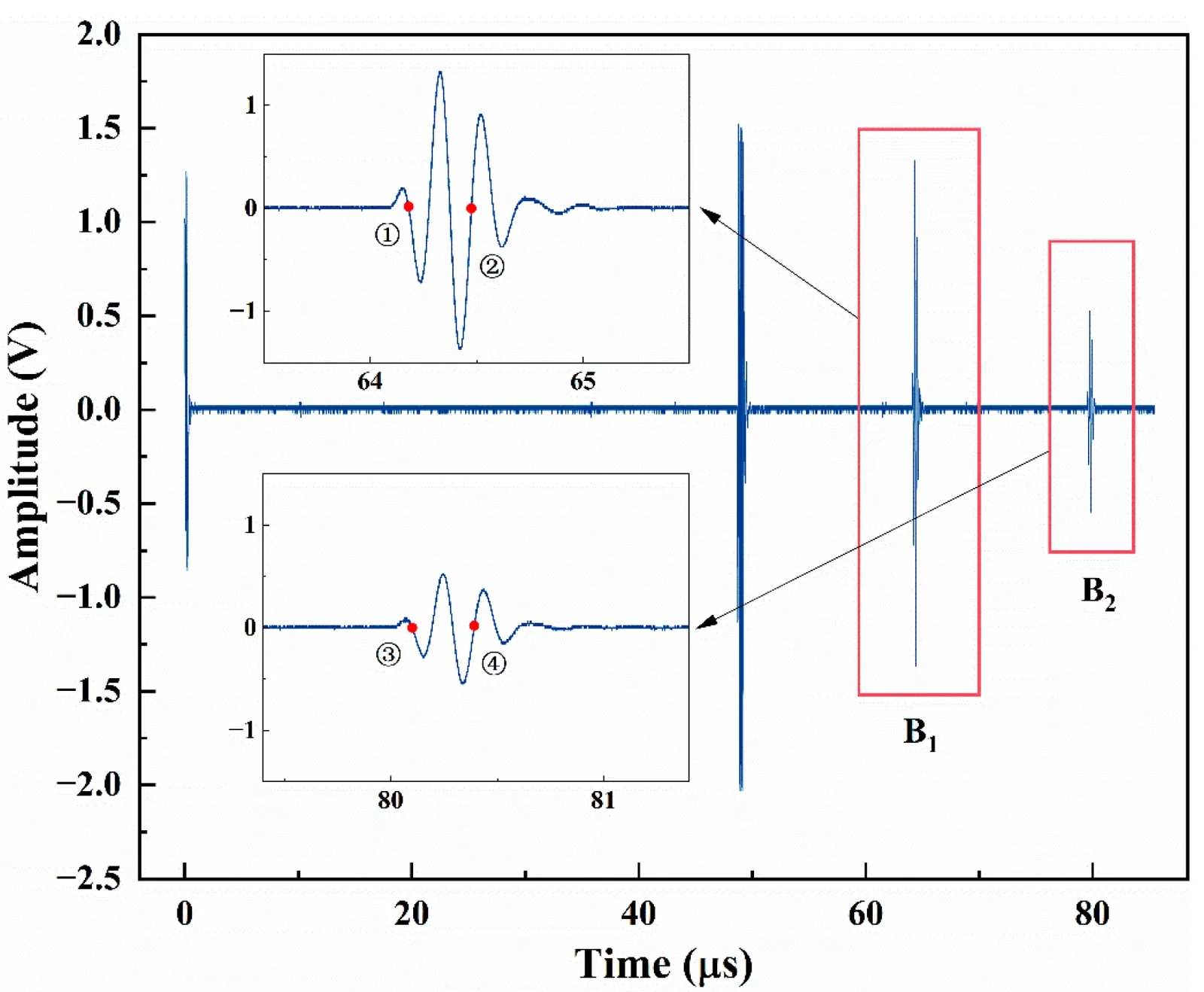
Typical ultrasonic echo signals recorded from the LAS glass ceramic sample.

Based on the above analysis, the water path *H* was fixed at 35 mm and the gain at 31 dB. A sampling rate of 1.25 GS/s was adopted to enhance the temporal resolution of the ultrasonic waveforms. Ultrasonic echo signals encompassing the front surface echo (S_F_), primary bottom echo B_1_, and secondary bottom echo B_2_ of the sample were acquired within a single transmit–receive cycle. Potential noise interference on ultrasonic echo signals was mitigated by three measures in this study, as follows: (1) a double-shielded waterproof cable with acoustic impedance matching characteristics was employed; (2) upper and lower cutoff frequencies were configured on the JSR DPR300 pulser–receiver panel to pass only the target-signal frequency band and suppress out-of-band noise for a better signal-to-noise ratio; and (3) a 10-point moving-average filter was applied for smoothing and denoising the raw signals. The ultrasonic echo signal of the sample is presented in Figure 8.

LAS glass ceramics consist of crystalline phases and a glass matrix. Despite their complex structure, they exhibit excellent structural homogeneity. As a result, ultrasonic waves present very low attenuation when propagating through LAS glass ceramics, and the ultrasonic pulse receiver can effectively detect the secondary bottom echo B_2_. As illustrated in Figure 8, the B_1_ and B_2_ signals from LAS glass ceramics show pronounced waveform similarity. Accordingly, the correlation method is employed to estimate the time interval between B_1_ and B_2_; i.e., the ultrasonic TOF. Denoting signal B_1_ by *s*_1_(*t*) and signal B_2_ by *s*_2_(*t*), the correlation coefficient of the two echo signals is calculated as

(7) $$R=\frac{\sum s_{1}(i)s_{2}(i)-\sum s_{1}(i)\sum s_{2}(i)/n}{{\left\{[\sum s_{1}^{2}(i)-{\left(\sum s_{1}(i)\right)}^{2}/n][\sum s_{2}^{2}(i)-{\left(\sum s_{2}(i)\right)}^{2}/n]\right\}}^{1/2}},i=1,2,\ldots ,n.,$$

where *n* represents the computation length of the signal array and *i* denotes the position index within the array.

Following the correlation coefficient calculation principle, the full ultrasonic signal containing both B_1_ and B_2_ is denoted as *s*(*i*). The signal segment between point ① and point ② on echo B_1_ is extracted as *s*_1_(*i*), and the segment between point ③ and point ④ on echo B_2_ is extracted as *s*_2_(*i*). The correlation coefficient between *s*_1_(*i*) and *s*_2_(*i*) is calculated via Equation (7), and the data point corresponding to the peak correlation coefficient is identified and denoted as *m*. The position of data point *m* is converted to the corresponding time using the sampling rate, from which the TOF is derived.

(5) *c*_L_ calculation

*c*_L_ was obtained by substituting the measured *d* in step (1) and the corresponding TOF (∆*t*) determined in step (4) into Equation (2). The *c*_L_ values of the above 17 samples at 25 °C are given in Table 4.

### 4.2. CTE Calibration

After completing the *c*_L_ measurements and preparing the CTE calibration samples, the *α*_m_ (0–50 °C) was calibrated. The main steps were as follows.

(1) The sample was dried and cleaned, and *L*_0_ was measured at 20 °C (~50 mm with a precision of 0.01 mm).
(2) The sample was placed in the laser dilatometer and left to stand for 30 min.
(3) The sample was cooled to 0 °C using liquid nitrogen and held for 10 min, and then heated to 52 °C at a heating rate of 1 °C/min.
(4) The CTE test software (Linseis Dilatometer Measurement, version 2.00) recorded Δ*L* with *T*; that is, the amount of expansion.
(5) The (Δ*L*/*L*_0_)-*T* data from 0 to 50 °C was processed to calculate *α*_m_ (0–50 °C).

**Figure 9:**
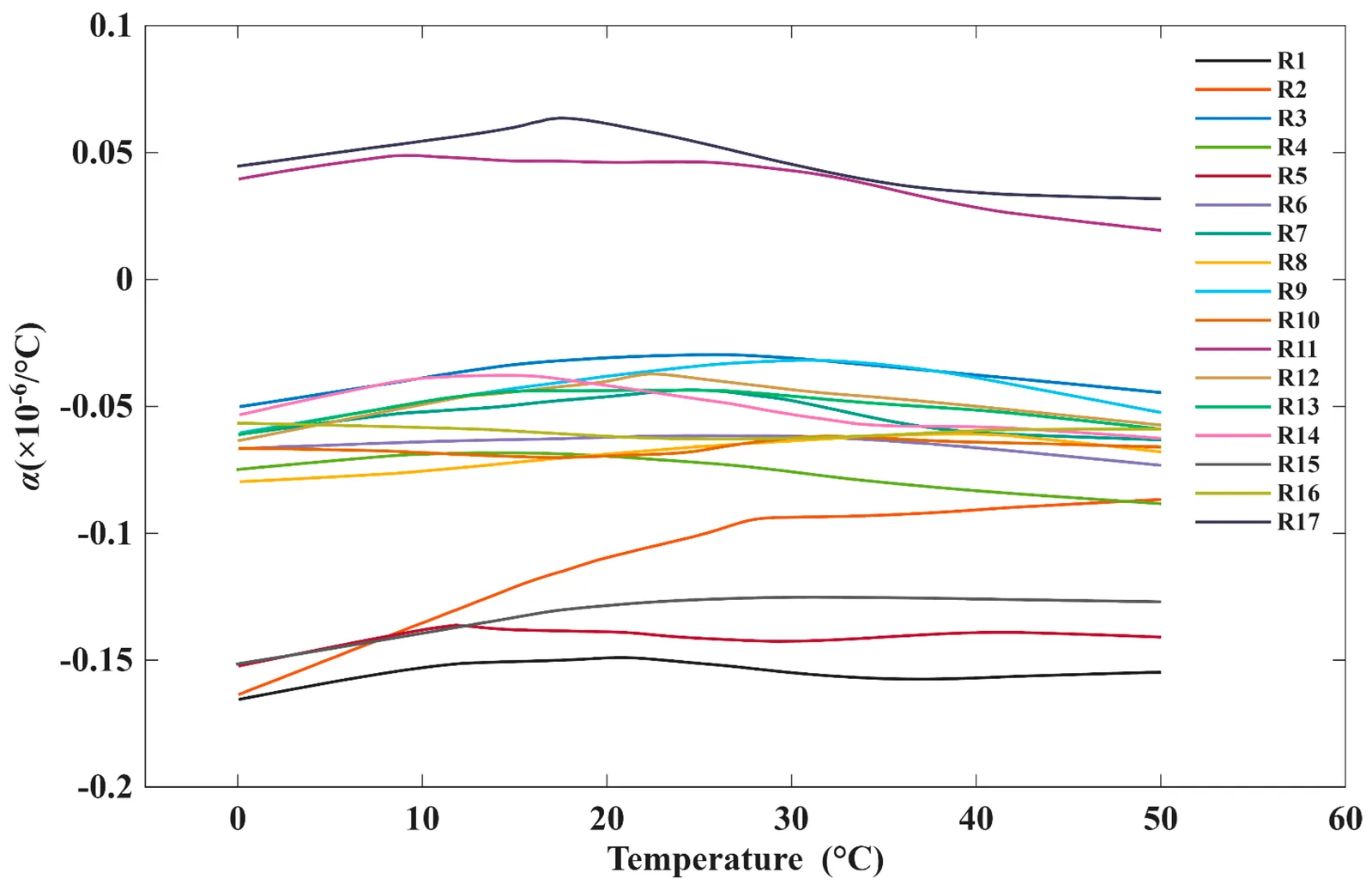
The calibration curves of the coefficient of thermal expansion for 17 LAS glass ceramic samples.

The software measured Δ*L*/*L*_0_ vs. *T* and produced variation curves for the instantaneous coefficient of linear expansion (*α*) with *T* in the range 0–50 °C (Figure 9). The calculated *α*_m_ (0–50 °C) values are given in Table 5.

### 4.3. Development of Ultrasonic CTE Measurement Model and Measurement Uncertainty Analysis

**Figure 10:**
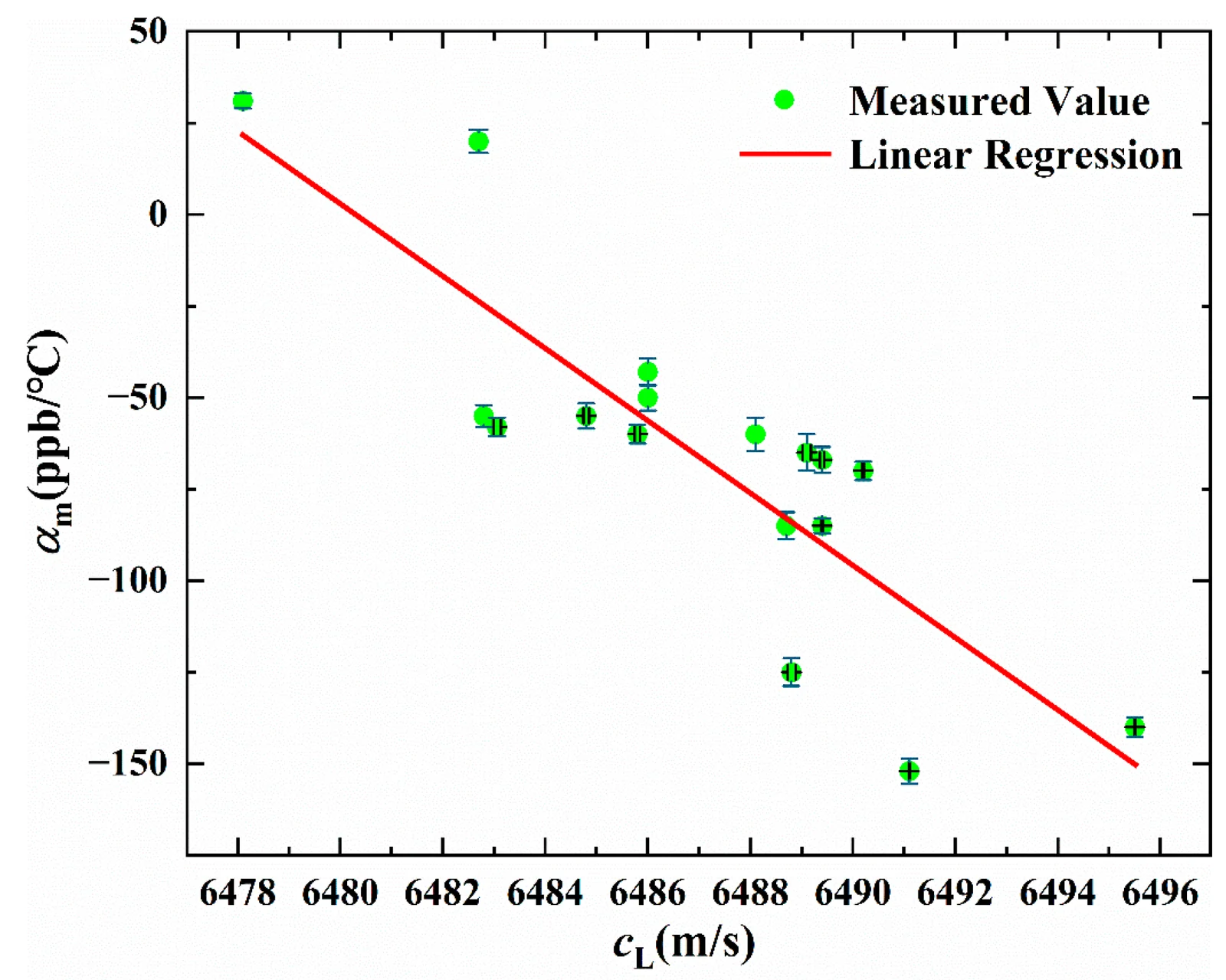
The relationship between *c*_L_ and *α*_m_ of the LAS glass ceramic samples. Error bars represent the standard deviation (SD) of repeated measurements.

Upon completion of ultrasonic velocity measurements and CTE calibration for all samples, the standard deviations of ultrasonic velocity measurements and CTE calibration were lower than 0.08 m/s and 3.7 ppb/°C, respectively. Both values are at relatively low levels, which provides a favorable basis for model construction. Accordingly, a fitting analysis of the relationship between *α*_m_ and *c*_L_ was performed on the LAS glass ceramic samples to establish an experimental model for CTE characterization via longitudinal wave velocity. The established model results with standard deviation error bars are presented in Figure 10.

The linear regression equation fitted to the experimental data of *c*_L_ and *α*_m_ using the least-squares method is expressed as

(8) $$\alpha_{m}=-9.75736c_{L}+63230.48,$$

Analysis of the fitted model yields a Pearson correlation coefficient of −0.84303 and a coefficient of determination of 0.7107. These values confirm a negative correlation between *c*_L_ and *α*_m_ in LAS glass ceramics and verify that the model achieves favorable fitting performance.

Based on the above model and the knowledge related to measurement uncertainty, the uncertainty of ultrasonic CTE measurement mainly includes the uncertainty related to the *c*_L_ value and the uncertainty related to the CTE calibration value.

(1) The *c*_L_ measurement uncertainty includes the thickness standard uncertainty *u*(*d*) and the TOF standard uncertainty *u*(Δ*t*). The S3 sample was analyzed as an example, and the synthetic standard uncertainty of *c*_L_ can be expressed as
  (9) $$u_{c}(c_{L})=c_{L}\times \sqrt{{\left(\frac{u(d)}{d}\right)}^{2}+{\left(\frac{u(\Delta t)}{\Delta t}\right)}^{2}},$$
  where both *u*(*d*) and *u*(Δ*t*) include class A and class B uncertainties. The analysis yielded synthetic standard uncertainties of 0.0004 mm and 0.00115 μs for *d* and Δ*t*, respectively. After substituting *c*_L_ and *d*, *u*_c_(*c*_L_) was calculated as 0.49 m/s.
(2) The sources of uncertainty in CTE calibration mainly include (1) the repeatability of the CTE measurement *u*(*α*), (2) the standard uncertainty of the initial length measurement *u*(*L*_0_), (3) the standard uncertainty of the ∆*T* measurement *u*(∆*T*), (4) the standard uncertainty of the ∆*L* measurement *u*(∆*L*), and (5) the uncertainty introduced by numerical modification *u*(*z*). Among these uncertainties, *u*(*L*_0_), *u*(∆*T*), *u*(∆*L*), and *u*(*z*) are analyzed via class B evaluation, and *u*(*α*) is analyzed via class A evaluation. The S3 sample was analyzed as an example. The relevant values of each uncertainty component are listed in Table 6, and the synthetic standard uncertainty of *α*_m_ (*u*_c_(*α*_m_)) was determined to be 1.66 ppb/°C. It can be found that *u*_c_(*α*_m_) mainly depends on *u*_A_(*α*_m_) and *u*(∆*L*). Both of these uncertainty values decrease when *L*_0_ increases, indicating a significant effort to improve calibration precision.
(3) From Equation (8), *α*_m_ is proportional to *c*_L_ with a scale factor of 9.75736 ppb/°C/(m/s). Thus, the uncertainty of the predicted CTE using *c*_L_ calculated from the uncertainty transfer equation is
  (10) $$u^{*}(\alpha_{m})=9.75736u_{c}(c_{L})\approx 4.78\;\mathrm{ppb}/℃.$$
  The uncertainty of ultrasonic nondestructive CTE testing should include the uncertainty of CTE calibration *u*_c_(α_m_) and the uncertainty caused by temperature fluctuations *u*_T_, where *u*_T_ ≈ 0.84 ppb/°C, from which the uncertainty was calculated to be
  (11) $$u_{p}(\alpha_{m})=\sqrt{{\left[u^{*}(\alpha_{m})\right]}^{2}+{\left[u_{c}(\alpha_{m})\right]}^{2}+{u_{T}}^{2}}\approx 5.13\;\mathrm{ppb}/℃.$$
  A coverage factor *k*_p_ = 2 was adopted, which corresponds to a 95% confidence probability for the expanded uncertainty. The expanded uncertainty of the ultrasonic αm measurement is calculated as
  (12) $$U(\alpha_{m})=k_{p}u_{p}(\alpha_{m})=10.26\;\mathrm{ppb}/℃.$$

The analysis indicates that the overall uncertainty level is primarily dominated by the ultrasonic velocity measurement accuracy and the CTE calibration accuracy. Further optimization can be implemented in two aspects; namely, improving the ultrasonic velocity measurement precision and optimizing the coefficients of the ultrasonic CTE experimental model, and enhancing the CTE calibration accuracy. According to the identified uncertainty sources, the measurement uncertainty of the model can be further reduced by improving the accuracies of ultrasonic velocity measurement and CTE calibration, fulfilling the demand for high-precision nondestructive inspection of CTE and its uniformity in large LAS glass ceramics.

## 5. Conclusions

In this paper, a UIPR method for measuring the CTE of LAS glass ceramics was proposed. High-precision *c*_L_ measurements were performed using a correlation algorithm, and the relationship between *α*_m_ (0–50 °C) and *c*_L_ (25 °C) of LAS glass ceramics was investigated. Some meaningful conclusions can be drawn.

(1) The ultrasonic CTE measurement model was established experimentally using the *c*_L_ values and calibrated *α*_m_ values (0–50 °C) of samples with distinctly different CTE levels. The ultrasonic velocity measurement performance of different ultrasonic transducer models was compared via both finite element simulations and physical experiments. On this basis, the model was fitted using experimental ultrasonic velocity data and calibrated CTE values of LAS glass ceramics with pronounced CTE differences, enabling high-precision CTE prediction with an uncertainty of 4.78 ppb/°C.
(2) The proposed ultrasonic method achieves a measurement uncertainty of 10.26 ppb/°C for the CTE determination in LAS glass ceramics.

By establishing a CTE experimental model based on the high-precision UIPR system, the proposed approach offers the combined advantages of nondestructive testing and cost efficiency. It can provide a novel testing solution for full-aperture, high-precision, rapid, and nondestructive measurement of CTE and its uniformity for LAS glass ceramics. Nevertheless, this method presents certain limitations. For example, it imposes high requirements on the regularity of the tested samples; furthermore, constrained by the dimensions of commercial ultrasonic transducers, the spatial resolution of the measurement remains to be further improved. In addition, the established complete uncertainty analysis system can provide directional guidance for the subsequent development of high-performance measurement devices.

Recent developments [24] in fractional modeling have provided alternative frameworks for describing thermal transport in advanced materials, particularly where anomalous diffusion, nonlocal effects, or memory effects are involved. While the present study is based on experimental measurements, extending the current analysis to fractional-order formulations could be an interesting and meaningful direction for future research.

## Figures and Tables

**Table 1:** Main parameters of the prepared samples for CTE calibration.

Parameters	Values
Density (g/cm^3^)	2.53
Young’s modulus (GPa)	90.3
Poisson’s ratio	0.24
Mean CTE(0–50 °C) (ppb/°C)	−160~50
Flatness	0.5*λ*
Parallelism (′)	2
End-face verticality (′)	1
Diameter (mm)	6 ± 0.5
Thickness (mm)	$50_{-0.1}^{0}$

**Table 2:** Model parameter settings.

Material Layer	Acoustic Velocity (m/s)	Density (g/cm^3^)	Young’s Modulus (GPa)	Poisson’s Ratio
Water	1500.0	1	-	-
LAS glass ceramics	6486.0	2.53	90.3	0.24

**Table 3:** Test results of simulated ultrasonic velocity accuracy and apparent signal attenuation characteristics for different transducers.

Frequency (MHz) –Diameter (mm)	Simulated Ultrasonic Velocity (m/s)	Deviation from the Theoretical Ultrasonic Velocity (%)	Primary Bottom Echo Amplitude (MPa)	Secondary Bottom Echo Amplitude (MPa)	Apparent Attenuation (dB)
3.5–13	6488.7	0.0420	0.15420	0.10028	−3.7
5–13	6484.1	0.0290	0.15796	0.10292	−3.7
5–10	6485.9	0.0015	0.15137	0.09608	−3.9
7.5–13	6481.5	0.0690	0.15695	0.09886	−4.0
10–6	6477.4	0.1300	0.12189	0.05431	−7.0
10–10	6480.4	0.0860	0.13520	0.06886	−5.9
15–6	6473.7	0.1900	0.13406	0.05549	−7.7

**Table 4:** The measurement results of the *c*_L_ of the LAS glass ceramic samples.

Serial Number	*d* (mm)	TOF (μs)	*c*_L_ (m/s)
S1	49.740	15.3256	6491.1
S2	49.740	15.3312	6488.7
S3	49.740	15.3376	6486.0
S4	49.740	15.3296	6489.4
S5	49.740	15.3152	6495.5
S6	49.741	15.3280	6490.2
S7	49.741	15.3384	6485.8
S8	50.005	15.4112	6489.4
S9	50.006	15.4197	6486.0
S10	50.005	15.4120	6489.1
S11	50.005	15.4272	6482.7
S12	50.006	15.4224	6484.8
S13	50.006	15.4272	6482.8
S14	50.005	15.4144	6488.1
S15	49.990	15.4016	6488.8
S16	49.990	15.4156	6483.1
S17	49.990	15.4260	6478.1

**Table 5:** The measurement results of the *α*_m_ (0–50 °C) of different LAS glass ceramic samples.

Serial Number	*L*_0_ (mm)	α_m_ (0–50 °C) (ppb/°C)
R1	49.64	−152
R2	49.63	−85
R3	49.63	−43
R4	49.64	−85
R5	49.64	−140
R6	49.64	−70
R7	49.63	−60
R8	49.79	−67
R9	49.79	−50
R10	49.79	−65
R11	49.80	20
R12	49.79	−55
R13	49.79	−55
R14	49.79	−60
R15	49.81	−125
R16	49.81	−58
R17	49.81	31

**Table 6:** Uncertainty analysis results of CTE calibration.

Analysis Method	Source	Value	Sensitivity Factor	Contribution
Class A	*u*_A_(*α*_m_)	1.5 ppb/℃	1	1.5 ppb/°C
Class B	*u*(∆*L*)	0.0017 μm	1/(*L*_0_Δ*T*)	0.69 ppb/°C
	*u*(∆*T*)	0.2 °C	*α*_m_/Δ*T*	0.16 ppb/°C
	*u*(*L*_0_)	0.003 mm	*α*_m_/*L*_0_	0.0024 ppb/°C
—	*u*(*z*)	0.03 ppb/℃	1	0.03 ppb/°C

## Data Availability

The original contributions presented in this study are included in the article. Further inquiries can be directed to the corresponding authors.
